# Non-*pylori Helicobacters* (NHPHs) Induce Shifts in Gastric Microbiota in *Helicobacter pylori*-Infected Patients

**DOI:** 10.3389/fmicb.2017.01038

**Published:** 2017-06-08

**Authors:** Xianhui Peng, Liya Zhou, Yanan Gong, Zhiqiang Song, Lihua He, Sanren Lin, Jianzhong Zhang

**Affiliations:** ^1^State Key Laboratory of Infectious Disease Prevention and Control, Collaborative Innovation Center for Diagnosis and Treatment of Infectious Diseases, National Institute for Communicable Disease Control and Prevention, Chinese Center for Disease Control and PreventionBeijing, China; ^2^Department of Gastroenterology, Peking University Third HospitalBeijing, China

**Keywords:** non-*pylori Helicobacter*s (NHPHs), gastric microbiota, *Helicobacter pylori*-infected, coinfection, 16S rRNA gene sequencing

## Abstract

To explore the effects of gastric non-*H. pylori Helicobacter* species(NHPH) on the structure and potential function of gastric microbiota, we employed 16S rRNA gene sequencing on 164 gastric biopsy specimens from NHPH (*H. suis, H. felis, H. salomonis*) /*H. pylori* coinfection individuals, *H. pylori* monoinfection individuals and healthy controls. The results demonstrated that marked structural and functional variations between *H. pylori* mono- and coinfection samples (HPHS, HPHF, HPHM). The changes in bacterial structure induced by NHPH are mainly attributed to their ability of gastric acid secretion inhibition as well as bacterial chemotaxis. Both the HPHS and HPHF groups showed significant increases in phylotype richness and significant decreases in β diversity, but this trend was not found in HPHM group. Regarding the top five phyla and top thirty-five genera, the HPHS and HPHF groups had similar variation trends in relative abundance. The increased relative abundance levels of the genera *Vibrio, Pseudoalteromonas, Photobacterium*, and *Clostridium* were associated with increases in predicted signal transduction/metabolic pathways among the three coinfection groups. The relative abundance levels of bacteria involved in the formation of *N*-nitroso compounds were significantly decreased in the HPHS and HPHF groups (e.g., *Streptococcus, Neisseria, Haemophilus, Veillonella, Clostridium, etc.*). The significantly decreased relative abundance levels of the phyla *Firmicutes* and *Bacteroidetes* in the HPHS and HPHF groups were associated with the observed increases in predicted lipid metabolism pathways. The results in this study implied that NHPH can arouse the variation of structure and function of gastric microbiota, which may pave the way to further research on the pathogenesis of gastric diseases.

## Introduction

Progress in sequencing technology and biological information analysis methods has enhanced our knowledge of gastrointestinal microbiota independent of traditional culture methods. The human stomach was long erroneously assumed to be sterile because of the gastric acid barrier. However, the discovery of *Helicobacter pylori* dispelled this dogma and opened the field for exploring the gastric microbiome. Increasing evidence suggests that the stomach possess a distinct and complex ecosystem inhabited by a wide variety of bacteria. Homeostasis of the stomach microecology is crucial for maintaining health, and its perturbation is considered a trigger of various stomach diseases (He et al., 2016). *H. pylori*, as the most relevant inhabitant, plays an important role in altering the gastric microenvironment by inhibiting acid secretion, leading to an imbalance in hormones, which might facilitate transient flora survival and colonization on the gastric epithelium, with subsequent disruption of the established gastric microbiota (Atherton and Blaser, 2009). Limited research (Bik et al., 2006; Stearns et al., 2011; Schulz et al., 2016; Sung et al., 2016) has led to a consensus that the presence of *H. pylori* dramatically affects species richness and the evenness of the gastric microbiota and does not change taxonomic diversity. The investigation of the human gastric microbiota is still in its infancy, and the roles and functions of other bacteria should be further elucidated.

In addition to *H. pylori*, other members of the genus *Helicobacter*, with typical spiral morphology (non-*pylori Helicobacter*, NHPH), are also associated with a range of gastric disorders, especially mucosa-associated lymphoid tissue (MALT) lymphoma (Baele et al., 2009; Haesebrouck et al., 2009). More than 30 species of the genus *Helicobacter* have been described; these species have been primarily isolated from domestic animals, rodents and non-human primates and have zoonotic potential (Baele et al., 2009; Haesebrouck et al., 2009). Humans may acquire these infections through direct contact with these animals (Haesebrouck et al., 2009). Previous studies reported that the prevalence of NHPH infection in humans varies from 0.2 to 6% depending on the geographic region (Yakoob et al., 2012). Recently, a study conducted in China showed that almost 12% of patients with gastric diseases are co-infected with NHPH and *H. pylori* (Liu et al., 2015). Among these NHPH species, four species were reported as the predominant organisms in the human stomach: *H. suis, H. salomonis, H. felis*, and *H. bizzozeronii*. Although the fastidious nature of these organisms seriously hampers further research, recent advances in *in vitro* isolation have improved our knowledge of their pathogenicity. In particular, the genome sequences of the species *H. suis, H. felis*, and *H. bizzozeronii* have revealed their roles in gastric pathology. All NHPH species’ genomes possess similar genes involved in metabolism and chemotaxis. Comparative genomic analysis among *H. suis, H. felis*, and *H. pylori* showed significant differences in genes involved in metabolism and chemotaxis (Schott et al., 2011). In addition to the genes necessary for life in the stomach, *H. suis* and *H. felis* have wider metabolic flexibility in aspects of energy sources and the electron transport chain than *H. pylori*. Furthermore, the *H. suis* and *H. felis* genomes have many more methyl-accepting chemotaxis proteins (MCPs) than that of *H. pylori*; these proteins permit bacteria to respond to a wider spectrum of environmental signals. Recently, a study concerning interactions between *H. suis* and gastric parietal cells determined that this bacterium can cause a series of side effects, significant impairment of cell viability, abnormal gastric acid secretion and disruption of stomach homeostasis (Zhang et al., 2016). Another study reported that *H. felis*-inoculated Mongolian gerbils showed loss of parietal cells and apoptotic loss of gastric epithelial cells (De Bock et al., 2006). In addition, it has been suggested that *H. felis* can switch off acid secretion, resulting in an increase in the pH of the gastric microenvironment. *H. pylori, H. suis, H. felis*, and *H. salomonis* equally belong to the genus *Helicobacter*, but notable differences exist in their complete genomes and their pathogenicities. In previous studies, too much attention was paid to *H. pylori*’s effect on the gastric microbiota, neglecting the contributions of NHPH species. Whether and how these NHPH species’ colonization of the gastric mucosa influences the structure of the gastric microbiota, bacterial diversity, and its possible functional metabolism should be further investigated.

In this study, we investigated the effects of NHPH species (*H. suis, H. felis, or H. salomonis*) and *H. pylori* coinfection on microbiota of gastric mucosa specimens from individuals undergoing gastroduodenoscopy by applying 16S rRNA gene deep sequencing. At the same time, the NHPH species-specific influences on the structure of the gastric microbiota were indirectly characterized by comparing variations of bacteria community between *H. pylori* mono- and coinfection groups. In addition, we predicted gene functions and metabolic pathways among different groups and found the striking changes in metabolic pathways caused by NHPH.

## Materials and Methods

### Study Subjects and Gastric Specimen Collection

From March 2014 to April 2015, a total of 1,500 gastric biopsy specimens were collected from individuals undergoing gastroduodenoscopy at Peking University Third Hospital. We excluded those subjects who received antibiotics, probiotics, proton pump inhibitors or H2 receptor antagonists within one month of specimen collection in our study. All samples were required for the rapid urease test (RUT), and species were further verified using gastric *Helicobacter* species-specific primers (Joosten et al., 2013; Liu et al., 2015). The final 164 cases were enrolled in this study and were classified into five groups: *H. pylori*/*H. suis* (+/+) group (HPHS, 39 cases), *H. pylori/H. felis* (+/+) group (HPHF,31 cases), *H. pylori/H. salomonis* (+/+) group (HPHM, 28 cases), *H. pylori* (+) group (HP, 33 cases) and control (*H. pylori*–, *H. suis*–, *H. felis*–, *H. salomonis*–) group (CT, 33 cases).

All gastric samples were preserved in RNAlater (QIAGEN, Germany), shipped on dry ice and stored at -80°C. This study was approved by the Ethics Committee of the National Institute for Communicable Disease Control and Prevention, Chinese Center for Disease Control. Written informed consent was obtained from all patients.

### Bacterial Genomic DNA Extraction

Each gastric biopsy specimen was homogenized in 1 mL of normal saline, and the homogenate was centrifuged to retain the pellet. Genomic DNA was extracted from the pellet with a QIAamp DNA Mini Kit (QIAGEN, Germany) following the manufacturer’s protocol for pathogenic genomes. To increase total DNA yields, lysozyme (Sangon Biotech, China) was added to the lysis buffer to promote the lysis of Gram-positive bacteria. The concentration and purity of genomic DNA were measured with a NanoDrop ND-1000 (Thermo Fisher Scientific, United States). The total genomic DNA was stored at –80°C prior to use.

### 16S rRNA Sequencing

The V3–V4 hypervariable regions of the 16S rRNA gene were amplified using a universal primer set (341F: CCTAYGGGRBGCASCAG, 806R: GGACTACNNGGGTATCTAAT) with a barcode. All template DNAs were normalized to the same concentration. PCRs were performed with Phusion High-Fidelity PCR Master Mix (New England Biolabs, United States). PCR products (approximately 500 bp) were separated by electrophoresis in agarose gels (2%, w/v), purified with a QIAGEN Gel Extraction Kit (QIAGEN, Germany) and pooled at equal concentrations. Sequencing libraries were generated using a TruSeq^®^ DNA PCR-Free Sample Preparation Kit (Illumina, United States) following the manufacturer’s recommendations, and index codes were added. Library quality was assessed on the Qubit@ 2.0 Fluorometer (Thermo Scientific) and the Agilent Bioanalyzer 2100 system. The library was sequenced on an Illumina HiSeq 2500 platform (250-bp paired-end reads) at Novogene Bioinformatics Technology Co., Ltd. (Beijing, China).

### Data Analysis

Paired-end reads were split and truncated based on the unique barcoded-primer sequence of every sample. Paired-end reads of each sample were merged using FLASH version 1.2.7 (Magoč and Salzberg, 2011). After merging, the generated raw tags were filtered to obtain high-quality clean tags according to QIIME version 1.7.0 (Caporaso et al., 2010). Chimera sequences were removed using the UCHIME algorithm (Edgar et al., 2011) to yield clean tags for further analysis.

Sequence analysis was performed using UPARSE pipeline version 7.0.1001 (Edgar, 2013), and sequences with ≥97% similarity were assigned to the same operational taxonomic units (OTUs). Representative sequences for each OTU were screened for further annotation. RDP Classifier (Version 2.2) was used to annotate taxonomic information for each representative sequence based on the Greengenes 13_5 Database (DeSantis et al., 2006). OTU abundance information was normalized using a standard sequence number corresponding to the sample with the fewest sequences. Subsequent analyses of α and β diversity were performed based on this output-normalized data.

### Statistical Analyses

A non-parametric *t*-test adjusted for age and sex based on Monte Carlo permutations was performed to compare the α diversity indices between groups in R statistical software^1^. A PERMANOVA test was used to determine the influences of age, sex, and infection status on β diversity (Weighted UniFrac distance and Bray–Curtis distance). Testing for significantly different phyla and genera between the two groups was performed using Metastats software^2^. The number of permutations used to calculate the *P*-value (significance threshold = 0.05, false discovery rate threshold = 0.2) was set to 1,000. To explore the functional profiles of different groups, we predicted the Kyoto Encyclopedia of Genes and Genomes (KEGG) pathways of the gastric community using the online protocol of PICRUSt (Phylogenetic Investigation of Communities by Reconstruction of Unobserved States^3^ (Langille et al., 2013). An OTU table based on 16S data was selected for PICRUSt from the Greengenes reference data (version 13.5). The nearest sequenced taxon index (NSTI) was used to assess the accuracy of the predictions for gene function and KEGG pathway enrichment in the gastric bacterial community. Welch’s *t*-test was employed to analyze the significant differences in KEGG pathways between groups.

## Results

### Statistical Summaries of V3-V4 16SrRNA Gene Sequencing Results

In this study, we characterized the gastric bacterial microbiota with different *Helicobacter* species’ infective status via 16S rRNA amplicon Illumina HiSeq 2000 sequencing. A total of 11.6 million paired-end raw reads were generated from 164 samples. After quality control (filtering and chimera removal), 8.2 million effective reads (50 ± 11 thousand reads per sample on average) were used for the subsequent analysis. At 97% sequence identity, a total of 105,864 OTUs were obtained for further annotation. The rarefaction curve of all samples nearly reached a plateau phase for sequencing depth, implying that the amount of sequencing data was reasonable (Supplementary Figure S1). The Good’s coverage index of all samples ranged from 96.5 to 99.8% (mean ± SD, 98.9% ± 0.6065%) (Supplementary Table S1).

### Bacterial Diversity

**FIGURE 1:**
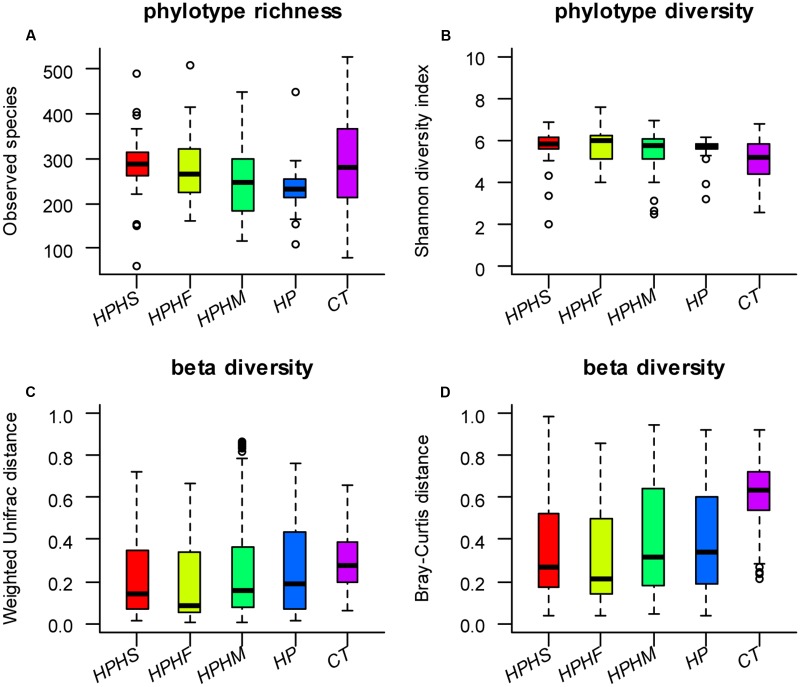
Distributions of α diversity indices (**A**: observed species index, **B**: Shannon diversity index) and β diversity indices (**C**: Weighted Unifrac distance based, **D**: Bray–Curtis distance based) among the five groups. HPHS: *Helicobacter suis* and *H. pylori* coinfection; HPHF: *H. felis* and *H. pylori* coinfection; HPHM: *H. salomonis* and *H. pylori* coinfection; HP: *H. pylori* monoinfection; CT: *Helicobacter*-negative.

To explore the bacterial community intra- and inter-variabilities among the five groups, we estimated a series of α diversity indices using the *t*-test and the Wilcoxon rank-sum test (**Table 1**). These indices, including a phylotype richness estimator (OTUs, observed species, Chao1, ACE) and a phylotype diversity estimator (Shannon, Simpson), are summarized in Supplementary Table S1. We found that the healthy control group had statistically significant increases in bacterial richness (OTUs, Chao1, ACE) and community diversity (Shannon, Simpson) compared with those of any of the other four groups (*P* < 0.0001, Monte Carlo test with 10,000 permutations). However, compared with HP group, only the HPHS and HPHF groups showed significant increases in phylotype richness (*P* < 0.05, Monte Carlo test with 10,000 permutations) and no significant differences in phylotype diversity were found in three coinfection groups. In addition, pairwise comparison among coinfection groups showed that significant difference was found only between the HPHS and HPHM groups. The distributions of observed species and Shannon diversity index among the five groups are displayed in **Figures 1A,B**. The statistical results of α diversity indices among the five groups are shown in **Table 1**.

**Table 1:** Comparisons of α diversity or β diversity indices among the five study groups.

Between-group	*P-*values (α Diversity Index)						*P-*values (β Diversity Index)	
	OTUs	Observed_ species	Chao1	ACE	Shannon	Simpson	Weighted Unifrac distance	Bray–Curtis distance
HP vs. CT	<0.0001	<0.0001	<0.0001	<0.0001	<0.0001	<0.0001	<0.0001	<0.0001
HPHS vs. CT	<0.0001	<0.0001	<0.0001	<0.0001	<0.0001	<0.0001	<0.0001	<0.0001
HPHF vs. CT	<0.0001	<0.0001	<0.0001	<0.0001	<0.0001	<0.0001	<0.0001	<0.0001
HPHM vs. CT	<0.0001	<0.0001	<0.0001	<0.0001	<0.0001	<0.0001	<0.0001	<0.0001
HPHS vs. HP	0.002	0.001	0.003	0.001	0.991	0.972	0.025	0.019
HPHF vs. HP	0.118	0.296	0.038	0.021	0.241	0.181	<0.0001	<0.0001
HPHM vs. HP	0.413	0.751	0.467	0.557	0.222	0.198	0.139	0.669
HPHS vs. HPHF	0.199	0.095	0.273	0.199	0.336	0.277	<0.0001	<0.0001
HPHS vs. HPHM	0.043	0.011	0.045	0.027	0.700	0.679	0.596	0.180
HPHF vs. HPHM	0.627	0.422	0.36	0.235	0.08	0.051	<0.0001	<0.0001

*P-values: Sex- and age-adjusted. HPHS: *H. suis* and *H. pylori* coinfection; HPHF: *H. felis* and *H. pylori* coinfection; HPHM: *H. salomonis* and *H. pylori* coinfection; HP: *H. pylori* monoinfection; CT: *Helicobacter*-negative.*

### Comparison of Gastric Microbiota at the Phylum and Genus Levels Among the Five Groups

**FIGURE 2:**
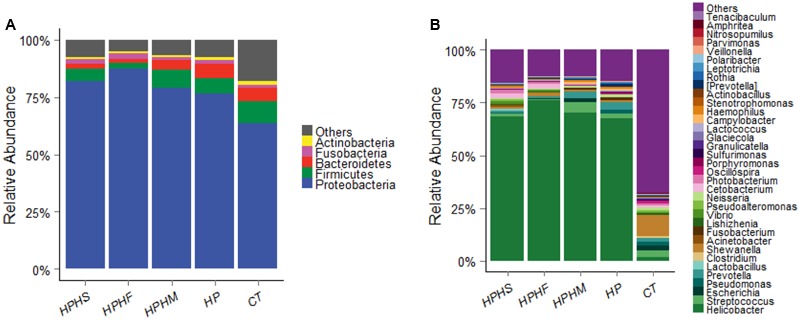
Relative abundance levels of bacterial phyla **(A)** and genera **(B)** in the gastric microbiota of the five groups. HPHS: *H. suis* and *H. pylori* coinfection; HPHF: *H. felis* and *H. pylori* coinfection; HPHM: *H. salomonis* and *H. pylori* coinfection; HP: *H. pylori* monoinfection; CT: *Helicobacter*-negative.

The predominant taxa of each group at the phylum and genus levels are shown in **Figure 2**. Comparison of the relative abundance among the five groups was performed by employing a metastats test (White et al., 2009), and the statistical results are summarized in **Table 2**. In particular, we analyzed the differences between the HP group and the other three coinfection groups. In general, compared with the relative abundance of the HP group, both the HPHS and HPHF groups had similar tendencies at the phylum and genus levels. At the phylum level, the five dominant phyla among the five groups were *Proteobacteria, Firmicutes, Bacteroidetes, Fusobacteria*, and *Actinobacteria*, accounting for 82–95% of the total microbiota. The HPHF group had significantly higher *Proteobacteria* (*P* = 0.0009) and significantly lower *Firmicutes, Bacteroidetes*, and *Actinobacteria* (*P* = 0.0009, 0.0009, and 0.0049, respectively) abundance levels than the HP group. Meanwhile, only *Bacteroidetes* showed a significant alteration from the HP group, with a decreased trend (*P* = 0.0019). No significant differences were observed between the HP and HPHM groups in the dominant phyla.

**Table 2:** Statistical significance at the phylum level among the five groups.

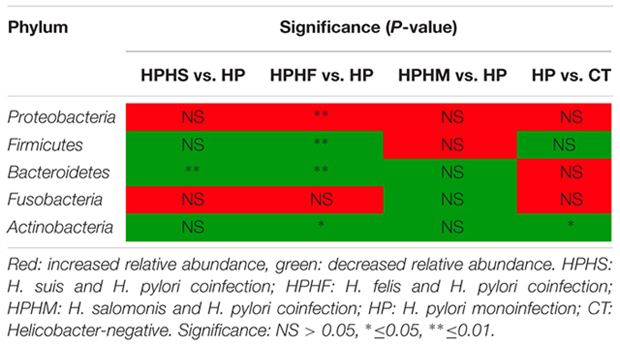

**FIGURE 3:**
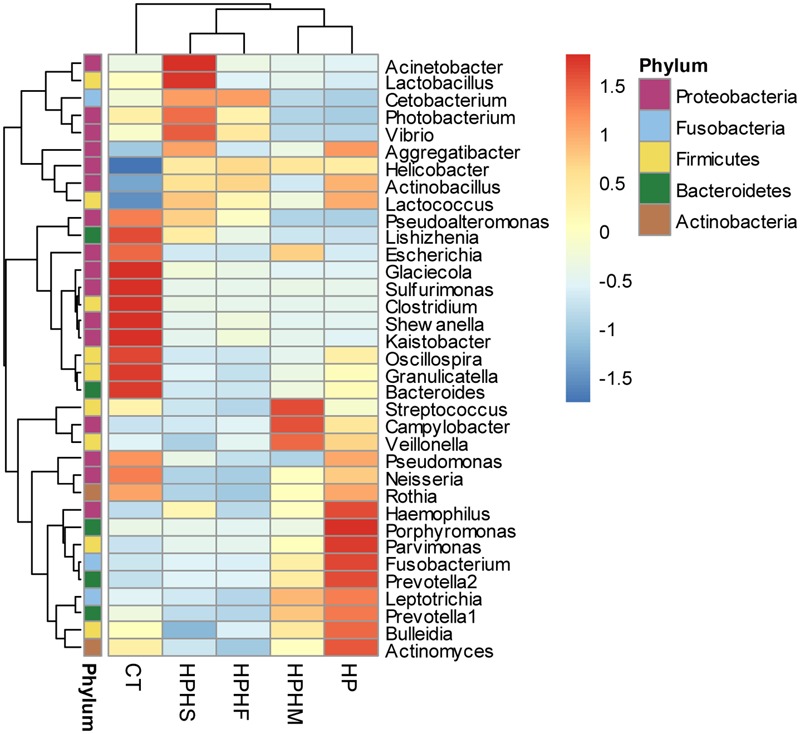
Heatmap of the relative abundance distributions of the genera. Red: up-regulation, blue: down-regulation. HPHS: *H. suis* and *H. pylori* coinfection; HPHF: *H. felis* and *H. pylori* coinfection; HPHM: *H. salomonis* and *H. pylori* coinfection; HP: *H. pylori* monoinfection; CT: *Helicobacter*-negative.

At the genus level, we focused on variations in the top 35 genera among the five groups. The gastric microbiota composition of the major genera for each group and the heatmap of variations in these genera among the different groups are shown in **Figure 3**. The results of the statistical analysis are shown in **Table 3**. The most dominant genus was *Helicobacter*, contributing 68.59, 75.98, 70.12, 67.60, and 1.88% of the gastric microbiota in the HPHS, HPHF, HPHM, HP, and CT groups, respectively. According to **Figures 2B, 3**, we found top 35 genera in five groups were enriched in top 5 phyla above. At the same time, distribution of the relative abundance on these genera in the CT group differed markedly from those found in the other four groups. However, HPHS and HPHF groups, HPHM and HP groups showed similar distribution of relative abundance on these genera. The relative abundance levels of 9 genera were significantly higher in the HSHP group than in the HP group (*P <* 0.05), including *Cetobacterium, Photobacterium, Vibrio, Pseudoalteromonas, Lishizhenia, Acinetobacter, Clostridium, Glaciecolai*, and *Kaistobacter.* In contrast, 13 genera were significantly less abundant in the HPHS group than in the HP group (*P* < 0.05), including *Prevotella, Neisseria, Fusobacterium*, [*Prevotella*], *Campylobacter, Oscillospira, Veillonella, Leptotrichia, Porphyromonas, Bulleidia, Aggregatibacter, Parvimonas*, and *Bacteroides.* The relative abundance levels of 8 genera were significantly more abundant in the HPHF group than in the HP group, including *Cetobacterium, Photobacterium, Vibrio, Pseudoalteromonas, Lactococcus, Lishizhenia, Glaciecola*, and *Kaistobacter.* In contrast, 21 genera were significantly less abundant in the HPHF group than in the HP group (*P* < 0.05), including *Streptococcus, Prevotella1, Escherichia, Neisseria, Fusobacterium, Haemophilus, Prevotella2, Rothia, Campylobacter, Oscillospira, Veillonella, Granulicatella, Leptotrichia, Porphyromonas, Actinobacillus, Bulleidia, Actinomyces, Aggregatibacter, Sulfurimonas, Parvimonas*, and *Bacteroides.* The relative abundance levels of 7 genera were significantly higher in the HPHM group than in the *H. pylori* group (*P* < 0.05), including *Cetobacterium, Photobacterium, Escherichia, Vibrio, Pseudoalteromonas, Lishizhenia*, and *Glaciecola.* In contrast, 6 genera were significantly less abundant in the HPHM group than in the *H. pylori* group (*P* < 0.05), including *Pseudomonas, Oscillospira, Porphyromonas, Actinobacillus, Aggregatibacter*, and *Sulfurimonas*.

**Table 3:** Statistical significance at the genus level among the five groups.

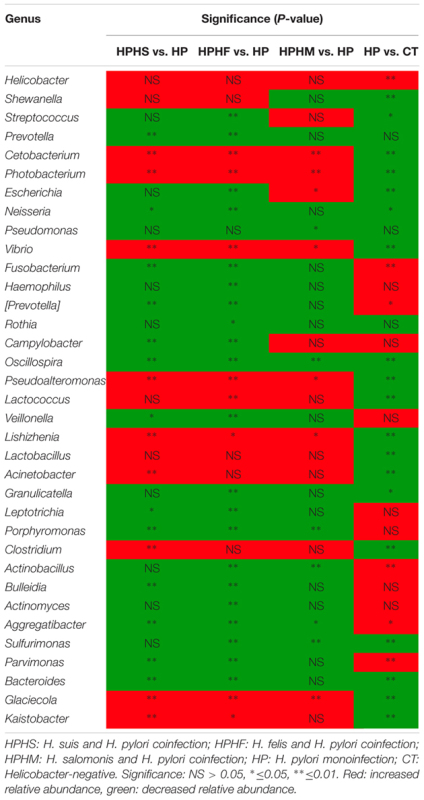

### Bacterial Community Structure

To compare variation in gastric community structure across the groups, Weighted Unifrac distance and Bray–Curtis distance indices were used to assess β diversity. A non-parametric test, the Mann-Whitney test based on Monte Carlo permutations (*n* = 10,000), was performed to determine differences in matrix distances between groups. Both the Weighted Unifrac and the Bray–Curtis distances revealed marked differences between the control group and any of the other four groups (*P* < 0.0001). In contrast, compared with the *H. pylori* group, although three coinfection groups displayed lower β diversities based on Weighted Unifrac distance (**Figure 1C**) and Bray–Curtis distance (**Figure 1D**), only the HPHS and HPHF groups showed significant differences (**Table 1**). Comparison among the three coinfection groups based on the Weighted Unifrac and Bray–Curtis distances also demonstrated significantly dissimilar communities apart from the HPHS and HPHM groups.

**FIGURE 4:**
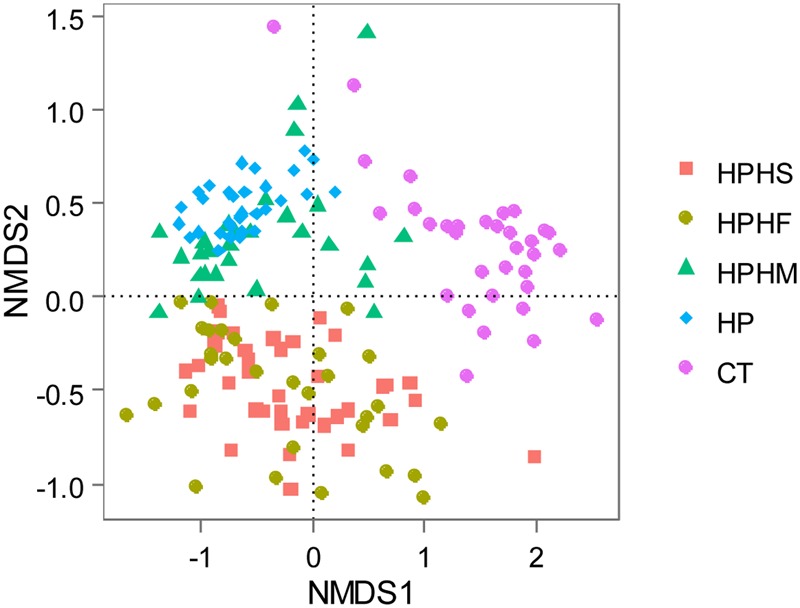
Non-metric Multidimensional Scaling (NMDS) distribution based on Weighted Unifrac distance. HPHS: *H. suis* and *H. pylori* coinfection; HPHF: *H. felis* and *H. pylori* coinfection; HPHM: *H. salomonis* and *H. pylori* coinfection; HP: *H. pylori* monoinfection; CT: *Helicobacter*-negative.

The Non-metric Multidimensional Scaling (NMDS) distribution based on Weighted Unifrac distance described the structures of the bacteria communities for all gastric samples (**Figure 4**). The distribution patterns showed obvious separation among the five groups, although overlapping microbiota were observed between HPHS and HPHF groups, HPHM and HP groups.

Following the above analysis of β diversity, we concluded that *H. salomonis* coinfection may only slightly change the microbiota pattern that *H. pylori* dominated. In contrast, infection with *H. pylori* coupled with *H. suis* or *H. felis* had a remarkable effect on the profile of the gastric bacterial community.

### Variations in Predicted Gastric Microbiota Function between the *H. pylori* Monoinfection Group and Coinfection Groups

**FIGURE 5:**
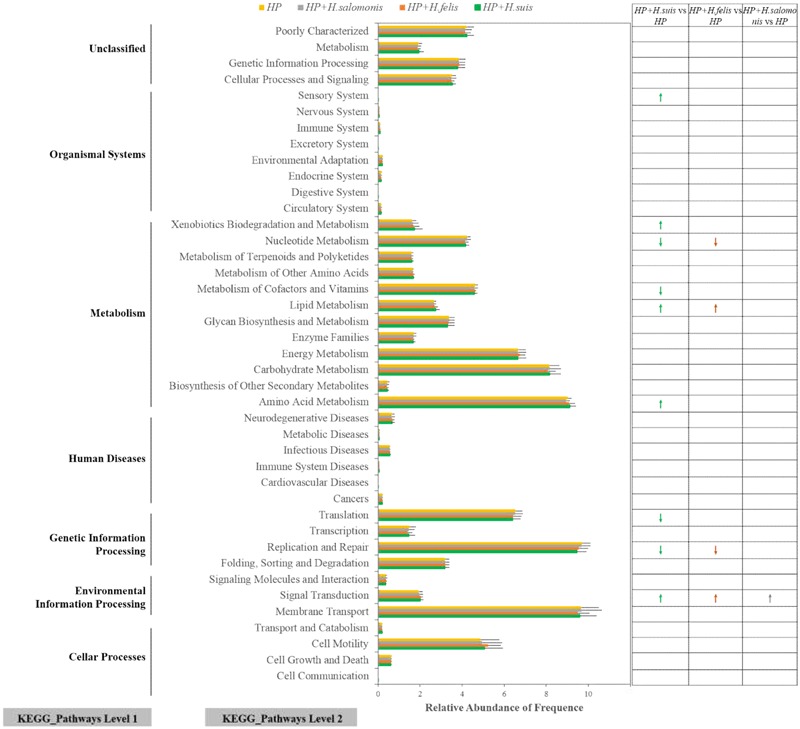
Relative abundance levels of predicted gene functions related to KEGG pathways at levels 1 and 2. Arrow: significant difference. Green: *HP* and *H. suis* coinfection, Ginger: HP and *H. felis* coinfection, Gray: HP and *H. salomonis* coinfection, Yellow: HP monoinfection.

Based on 16S rRNA gene sequencing data, PICRUSt was performed to predict gastric microbiota functions, which showed that similar gene functions among five groups in different infective states were categorized into 41 level 2 KEGG pathways. Before the prediction analysis, we found that the mean of the weighted NSTI for all samples was 0.041 ± 0.025, a value that was quite close to the earlier reported result in humans (NSTI mean ± SD = 0.03 ± 0.02), indicating that our study possesses a high accuracy of prediction for gene function and KEGG pathway enrichment in the gastric bacterial community. There were evident differences in the relative abundance levels of the most functional profiles between the *H. pylori* monoinfection group and the three coinfection groups (**Figure 5**). Although three coinfection groups presented similar functional compositions in the relative abundance levels, some differences among groups were obvious. We performed Welch’s *t*-test to compare the KEGG pathways with significant abundance levels between the *H. pylori* monoinfection group and the coinfection groups. We found that the abundance levels of nine KEGG pathways (Sensory System, Xenobiotics Biodegradation, and Metabolism, Nucleotide Metabolism, Metabolism of Cofactors and Vitamins, Lipid Metabolism, Amino Acid Metabolism, Translation, Replication and Repair, Signal Transduction) were significantly different between the HP and HPHS groups (Welch’s *t*-test, *P* < 0.05, **Figure 5**). Four KEGG pathways (Nucleotide Metabolism, Lipid Metabolism, Replication and Repair, Signal Transduction) significantly differed between the HP and HPHF groups (Welch’s *t*-test, *P* < 0.05, **Figure 5**). Only one KEGG pathway (Signal Transduction) was significantly different between the HP and HPHM groups (Welch’s *t*-test, *P* < 0.05, **Figure 5**).

## Discussion

This study is the first to explore the effects of NHPH species (*H. suis, H. felis, or H. salomonis*) and *H. pylori* coinfection on the stomach microbiota composition of gastric mucosa specimens from individuals undergoing gastroduodenoscopy by applying 16S rRNA gene deep sequencing. Simultaneously, the effects of NHPH species on the gastric microbiota were indirectly explained by comparing variations in gastric bacterial community structure between *H. pylori* monoinfection group and NHPH/*H. pylori* coinfection groups. In addition, we found that NHPH brought about the striking changes in metabolic pathways.

We found that the composition and diversity of the gastric microbiota in our study shifted among all five groups in a complicated fashion. We found that compared with the control group, *H. pylori*-positive groups (including *H. pylori* mono- and coinfection groups) possessed unique features in their gastric microbiota. These features involved significantly depleted microbial community α diversity, markedly different microbiota distributions and lower inter-individual variabilities. In accordance with previous reports (Maldonado-Contreras et al., 2011; Aviles-Jimenez et al., 2014), the gastric mucosa of *H. pylori*-positive individuals was highly dominated by this organism, concomitant with depletions in the relative abundance levels of other genera. In contrast to a previous study, the presence of *H. pylori* not only had a significant influence on species richness but also changed the taxonomic diversity in this study.

In contrast, there was relatively low divergence among the four *H. pylori*-positive groups. The coinfection groups also showed higher gastric community α diversity and lower inter-individual variability than the *H. pylori* monoinfection group. However, compared with the *H. pylori* group, only the HPHS and HPHF groups showed significant differences in the α and β diversity indices. The relative abundance levels of the predominant taxa in the global gastric microbiota of each group were altered markedly. The three coinfection groups appeared to maintain similar changes in the trend in the relative abundance levels of genera and phyla compared with the *H. pylori* monoinfection group, but exceptions emerged in the HPHM group. A possible explanation is that dual infection with *H. pylori* and other members of the *Helicobacter* species can cause variations in the gastric microbiota. The effects are highly species-specific; the entire profile of the gastric microbiota is dependent on the type of gastric NHPH species, which may promote or attenuate variation in *H. pylori*-predominated gastric microbiota. At a more detailed level, we paid close attention to the variations in the gastric core microbiota among groups, including the top 5 phyla (*Proteobacteria, Firmicutes, Bacteroidetes, Fusobacteria, and Actinobacteria*) and the top 35 genera. An analysis of the community compositions of the *H. pylori* mono- and coinfection groups revealed significant differences, primarily enrichment in the relative abundance levels of *Proteobacteria, Firmicutes, Bacteroidetes* and *Fusobacteria*. Genera that significantly increased in abundance among coinfection groups derived from the phylum *Proteobacteria*, while other genera, namely, from the phyla *Firmicutes, Bacteroidetes*, and *Fusobacteria*, significantly decreased in abundance. Intriguingly, the results of the comparison between the *H. pylori* monoinfection group and the *H. pylori*-negative control group were opposite those of the coinfection groups and the *H. pylori* monoinfection group in the genera with significant differences in relative abundance. These genera included *Cetobacterium, Photobacterium, Vibrio, Pseudoalteromonas, Lishizhenia, Aggregatibacter*, and *Glaciecola*. The results implied that the presence of NHPH species can significantly alter the structure of an *H. pylori-*dominated gastric bacterial community.

Gastric acid is a hostile territory for microbial colonization and overgrowth. However, acid secretion was perturbed after *H. pylori* infection. Intragastric pH alteration due to decreased acid secretion may facilitate survival of passenger bacteria from the oral mucosa, the upper-respiratory tract and the esophagus even if the bacteria cannot persist in the healthy stomach. Similarly, *H. suis* and *H. felis* can reportedly inhibit and even switch off gastric acid secretion in various ways. Therefore, we speculated that *H. suis* and *H. felis* may enhance inhibition of gastric acid secretion by *H. pylori*. This phenomenon explained our results, specifically that the α diversity (Shannon diversity index) of the gastric microbiota increased in the *H. pylori* monoinfection group, while higher increases in α diversity were found in the HPHS and HPHF groups. Additionally, no research has shown whether or how *H. salomonis* can change the surrounding gastric acid secretion. However, in line with the HPHS and HPHF groups, increased community richness in the gastric microbiota in the HPHM group indicated that *H. salomonis* may play a role in altering *H. pylori-*dominant gastric microbiota. Further exploration is needed to support this prediction. In addition to decreased acid secretion, *H. pylori, H. felis, H. suis, H. salomonis*, and other *Helicobacter* species can employ urease to produce ammonia and bicarbonate from urea, which serve as substrates for the surrounding associated bacterial growth (Williams et al., 1996).

Bacterial chemotaxis plays a major role in structuring microbial communities (Miller et al., 2009), affecting microbial activities and influencing various microbial interactions with related surroundings. Bacterial chemotaxis is controlled by a two-component system (TCS) whose core component is the so-called MCPs (Szurmant and Ordal, 2004; Kirby, 2009). A comparative whole-genome analysis elucidated that *H. suis* and *H. felis* differ from *H. pylori* by having wider metabolic flexibilities in terms of energy sources and electron transport chains and by harboring higher numbers of MCPs that allow them to respond to a wider spectrum of environmental signals. Furthermore, among the genera with increased relative abundance levels, *Vibrio, Pseudoalteromonas, Shewanella, Photobacterium*, and *Clostridium* have been reported to possess many chemoreceptors (Miller et al., 2009), which may enable the monitoring of their environments to detect chemicals over wide concentration ranges. Taken together, the above investigation can provide a good illustration of our PICRUSt prediction of the significant increases in metabolism pathways associated with signal transduction among the three coinfection groups. In addition, the genus *Acinetobacter* contains genes encoding photosensory receptor proteins, which have roles in regulating gene expression for phototactic responses and signaling processes (Nudel and Hellingwerf, 2015). The significant increase in *Acinetobacter* spp. detected in the HPHS group from gastric microbiota implies a potential utility for sensing environmental responses and cell signaling processes. PICRUSt analysis revealed that predicted gene functions associated with sensor metabolism pathways are significantly increased in the HPHS group.

*N*-nitroso compounds (NOCs) are potent carcinogens. NOC formation has been suggested to increase the risk of gastric cancer (Mowat et al., 2000; Dicksved et al., 2009), and some stomach bacteria are known to be involved in NOC formation. A pyrosequencing study stated that the proportion of non-*H. pylori* -NB was two times higher in the gastric cancer group than in a control group with same *H. pylori* status (Jo et al., 2016). In our study, the relative abundance levels of these alleged NBs were significantly decreased both in the HPHS and HPHF groups, including the genera *Streptococcus, Prevotella, Escherichia, Neisseria, Pseudomonas, Haemophilus, Campylobacter, Veillonella, Lactobacillus, Clostridium*, and *Bacteroides*. However, not all relatively abundant genera showed significant variations in the HPHM group. Based on the view above, we conclude that co-infected individuals in our study have a lower probability of developing gastric cancer than the *H. pylori* monoinfection group.

The phyla *Firmicutes* and *Bacteroidetes* are enriched with genes encoding many enzymes that regulate lipid metabolism, maintaining energy homeostasis in the host (Hwang et al., 2015). The significant decreases in *Firmicutes* and *Bacteroidetes* detected in the gastric microbiota in both the HPHS and HPHF groups indicate their potential utility in lipid metabolism. Comparative functional analysis with PICRUSt also demonstrated that predicted gene functions associated with carbohydrate and lipid metabolism pathways were significantly increased in the HPHS and HPHF groups.

## Author Contributions

JZ and XP designed experiments; XP, LH, LZ, and ZS carried out experiments; XP analyzed experimental results; XP analyzed sequencing data and developed analysis tools; Novogene Bioinformatics Technology Co., Ltd assisted with Illumina sequencing. XP wrote the manuscript; JZ modified the manuscript.

## Conflict of Interest Statement

The authors declare that the research was conducted in the absence of any commercial or financial relationships that could be construed as a potential conflict of interest.

## Footnotes

1 http://www.r-project.org/

2 http://metastats.cbcb.umd.edu/

3 http://picrust.github.io/picrust/
