# Supplementary material for: Non-pylori Helicobacters (NHPHs) Induce Shifts in Gastric Microbiota in Helicobacter pylori-Infected Patients
Source: Front Microbiol. 2017 Jun 8;8:1038. doi: 10.3389/fmicb.2017.01038 (PMC5462978; doi:10.3389/fmicb.2017.01038)
Supplement: Supplementary file 1 [file Data_Sheet_1.pdf]

Table S1 Summary of α diversity index.

Sample Name	OTUs	Observed_ species	Chao1	ACE	Shannon	Simpson	Goods_ coverage
HPHS0095	780	537	719.026	755.123	3.524	0.670	0.987
HPHS0096	500	300	462.983	505.087	1.624	0.304	0.991
HPHS0118	411	264	327.553	333.906	4.196	0.891	0.996
HPHS0187	758	437	654.277	719.287	2.405	0.475	0.987
HPHS0200	554	307	459.721	504.265	1.524	0.280	0.991
HPHS0209	679	361	664.738	718.138	2.006	0.395	0.988
HPHS0246	654	422	618.463	656.314	2.699	0.523	0.989
HPHS0274	575	345	455.584	485.885	1.905	0.366	0.992
HPHS0286	845	580	833.685	820.708	4.714	0.832	0.987
HPHS0329	165	135	151.533	159.985	2.133	0.566	0.998
HPHS0339	466	287	422.344	497.448	2.666	0.675	0.992
HPHS0347	511	310	492.283	498.501	1.915	0.362	0.992
HPHS0350	676	480	673.800	673.527	4.398	0.805	0.989
HPHS0362	734	446	689.140	745.661	2.616	0.499	0.987
HPHS0372	571	336	541.776	586.184	2.358	0.466	0.990
HPHS0488	625	332	558.542	601.258	1.802	0.342	0.990
HPHS0489	500	273	419.542	508.652	1.221	0.216	0.992
HPHS0508	497	293	513.521	527.908	1.489	0.277	0.991
HPHS0530	515	260	412.520	452.623	1.166	0.210	0.992
HPHS0590	869	547	791.660	821.004	3.000	0.530	0.986
HPHS0638	632	385	512.530	554.434	2.120	0.376	0.991
HPHS0639	676	401	576.066	648.939	2.060	0.389	0.989
HPHS0668	488	269	420.961	463.236	1.296	0.240	0.992
HPHS0701	567	280	511.122	552.519	1.049	0.188	0.990
HPHS0708	1161	627	1082.714	1174.483	2.952	0.531	0.980
HPHS0742	546	356	490.717	469.997	2.908	0.558	0.992
HPHS0745	1703	1218	1875.360	1979.577	6.126	0.918	0.966
HPHS0831	697	353	682.000	738.035	2.414	0.500	0.988
HPHS0850	581	266	560.525	634.279	1.026	0.180	0.990
HPHS0857	626	321	552.694	646.468	1.246	0.221	0.989
HPHS0902	538	242	442.025	455.154	0.911	0.169	0.992
HPHS0927	613	345	532.548	554.877	1.834	0.339	0.990
HPHS0975	1183	746	1031.207	1064.211	5.835	0.933	0.983
HPHS1318	539	266	454.019	552.632	0.944	0.166	0.991
HPHS1437	958	511	866.110	972.843	1.976	0.347	0.983
HPHS1495	529	336	528.500	596.158	2.125	0.411	0.990
HPHS1547	845	571	904.808	959.619	3.265	0.610	0.983
HPHS1563	721	523	746.897	698.109	5.659	0.946	0.989
HPHS1810	538	344	475.087	496.816	1.961	0.358	0.992
HPHF0168	436	247	350.000	391.509	1.276	0.230	0.994
HPHF0259	414	228	462.370	420.374	1.042	0.184	0.993
HPHF0292	312	173	284.000	283.155	1.090	0.213	0.995
HPHF0430	273	143	233.621	286.777	0.664	0.126	0.995
HPHF0434	471	268	441.776	518.350	1.315	0.233	0.992
HPHF0437	799	397	707.514	802.451	1.750	0.306	0.987
HPHF0440	302	145	291.714	299.624	0.584	0.102	0.995
HPHF0443	453	273	406.982	441.631	1.399	0.275	0.992
HPHF0444	500	297	448.682	504.603	1.956	0.410	0.991
HPHF0448	661	377	599.581	678.018	1.523	0.260	0.989
HPHF0456	686	391	724.915	756.749	1.829	0.328	0.987
HPHF0465	605	373	606.429	666.524	2.280	0.424	0.989
HPHF0467	542	285	435.492	510.150	1.365	0.248	0.991
HPHF0469	447	239	446.237	491.693	1.071	0.195	0.992
HPHF0479	1027	550	904.673	965.049	1.704	0.270	0.983
HPHF0516	523	328	495.354	530.195	1.303	0.221	0.991
HPHF0519	953	614	892.258	868.960	4.867	0.903	0.986
HPHF0525	844	456	822.176	912.033	1.888	0.347	0.984
HPHF0527	583	320	517.516	560.556	1.423	0.251	0.990
HPHF0570	615	388	525.274	596.144	1.985	0.380	0.990
HPHF0922	453	231	372.977	404.697	0.866	0.147	0.993
HPHF1012	471	250	437.957	484.771	1.368	0.267	0.992
HPHF1013	513	323	469.300	476.293	2.215	0.476	0.992
HPHF1084	472	344	593.918	638.862	3.786	0.822	0.990
HPHF1116	503	369	494.609	489.121	4.134	0.786	0.993
HPHF1125	361	231	328.683	350.170	2.184	0.508	0.994
HPHF1127	728	410	663.808	760.172	3.352	0.749	0.988
HPHF1195	842	492	882.231	986.973	1.868	0.324	0.983
HPHF1237	874	594	862.784	845.691	5.977	0.960	0.986
HPHF1238	590	343	670.308	710.166	1.688	0.305	0.988
HPHF1290	315	191	260.517	268.009	1.628	0.365	0.996
HPHM0483	317	202	288.059	300.032	1.781	0.394	0.995
HPHM0486	239	145	275.789	260.564	1.140	0.282	0.996
HPHM0525	311	218	334.400	361.379	0.961	0.167	0.994

HPHM0553	489	326	437.196	452.038	5.096	0.930	0.993
HPHM0704	326	181	235.737	259.999	1.808	0.509	0.996
HPHM0723	435	302	387.024	373.262	2.879	0.617	0.995
HPHM0741	599	466	522.097	543.200	3.421	0.638	0.993
HPHM0748	472	278	452.146	451.330	2.028	0.403	0.992
HPHM0757	297	174	263.062	289.172	1.177	0.240	0.995
HPHM0764	610	333	575.354	639.732	1.246	0.224	0.989
HPHM0768	562	331	521.122	509.869	2.886	0.568	0.991
HPHM0776	627	347	571.380	623.030	1.039	0.170	0.989
HPHM0777	608	333	578.565	630.734	1.407	0.260	0.989
HPHM0780	501	267	405.158	454.479	1.159	0.206	0.992
HPHM0784	299	160	241.375	229.737	0.842	0.150	0.996
HPHM0793	457	284	434.060	461.761	1.758	0.332	0.992
HPHM0794	511	281	443.018	481.869	1.275	0.229	0.992
HPHM0815	455	235	374.125	383.249	0.991	0.171	0.993
HPHM0819	1139	851	1038.987	1060.285	5.786	0.907	0.985
HPHM0908	548	378	550.971	601.800	3.176	0.606	0.990
HPHM0956	559	358	551.439	567.856	2.757	0.535	0.991
HPHM0960	563	361	442.128	477.396	4.117	0.821	0.993
HPHM1004	556	402	513.523	547.681	2.696	0.494	0.991
HPHM0012	464	234	313.222	348.938	1.120	0.197	0.994
HPHM0020	692	529	623.000	650.024	4.314	0.753	0.991
HPHM0024	502	243	387.383	429.988	1.158	0.211	0.993
HPHM0030	434	258	359.368	412.970	2.039	0.413	0.993
HPHM0031	530	318	486.808	473.972	1.879	0.352	0.992
HP0081	326	204	265.116	288.145	1.390	0.261	0.995
HP0093	409	254	394.622	418.980	1.582	0.312	0.993
HP0105	395	229	318.538	388.060	3.297	0.775	0.994
HP0115	398	236	343.312	368.856	1.300	0.248	0.994
HP0143	357	202	279.766	309.583	0.958	0.175	0.995
HP0498	672	518	715.010	773.760	5.167	0.927	0.987
HP0500	397	243	408.512	430.795	1.204	0.223	0.993
HP0528	330	208	293.000	322.702	0.998	0.178	0.995
HP0601	439	311	367.038	400.729	2.033	0.401	0.994
HP0630	950	459	718.517	766.790	2.748	0.543	0.987
HP0680	512	386	551.878	541.962	5.357	0.935	0.992
HP0689	243	130	168.897	181.018	0.563	0.096	0.997
HP0699	460	320	456.685	477.985	2.607	0.495	0.992
HP0706	400	263	402.216	379.481	1.550	0.291	0.994
HP0738	665	482	595.610	613.860	4.735	0.853	0.991
HP0797	594	467	556.034	545.634	5.940	0.958	0.994
HP0807	682	464	625.284	635.974	5.309	0.930	0.990
HP0812	517	344	449.000	470.828	3.132	0.720	0.992
HP1265	455	397	453.226	461.666	5.703	0.951	0.995
HP1334	349	204	287.133	323.564	0.933	0.164	0.995
HP1339	400	272	369.021	383.429	2.096	0.392	0.994
HP1384	420	263	339.393	391.049	2.856	0.589	0.994
HP1391	504	311	420.103	448.298	2.943	0.573	0.993
HP1397	352	193	270.467	295.749	0.776	0.134	0.995
HP1403	630	436	581.918	615.931	3.708	0.699	0.990
HP1406	534	352	493.000	524.042	1.971	0.380	0.991
HP1410	505	306	399.059	433.451	2.052	0.395	0.993
HP1415	471	322	419.225	443.861	2.300	0.448	0.993
HP1579	242	134	223.375	237.237	0.467	0.077	0.996
HP1690	469	289	404.098	444.447	1.552	0.283	0.993
HP1740	286	211	262.419	272.252	1.083	0.188	0.996
HP1761	404	255	352.382	383.916	1.685	0.323	0.993
HP1788	402	234	374.947	392.994	1.528	0.288	0.993
CT0348	1506	1237	1727.880	1718.327	5.908	0.863	0.971
CT0350	1556	1092	1447.857	1552.963	6.048	0.932	0.973
CT0351	1029	786	1103.462	1126.698	5.572	0.870	0.982
CT0352	1292	1075	1239.709	1267.309	6.627	0.929	0.984
CT0353	1601	1287	1939.954	2046.301	6.150	0.898	0.965
CT0357	1598	1286	1813.004	1915.097	5.864	0.899	0.967
CT0358	792	538	871.821	985.409	5.333	0.843	0.987
CT0360	1105	1105	1702.333	1832.019	6.156	0.939	0.968
CT0361	779	625	737.048	819.667	5.338	0.862	0.989
CT0365	1023	863	1043.277	1071.172	6.962	0.947	0.986
CT0368	933	722	1015.684	1051.138	4.946	0.886	0.982
CT0371	1369	878	1300.169	1336.835	6.151	0.948	0.978
CT0374	1138	894	1462.993	1485.856	5.712	0.911	0.975
CT0386	972	679	938.000	972.432	4.460	0.850	0.984
CT0388	1029	853	1014.817	1109.810	6.323	0.929	0.984
CT0389	1127	629	1033.634	1122.863	3.005	0.633	0.980
CT0396	1366	880	1348.742	1434.455	4.625	0.863	0.974
CT0397	696	474	673.396	748.695	4.286	0.809	0.988
CT0401	810	481	772.261	867.252	2.977	0.648	0.985

CT0402	1198	915	1414.333	1535.740	5.214	0.891	0.973
CT0405	1052	898	1041.415	1073.321	5.840	0.921	0.985
CT0407	1039	773	1115.400	1233.358	5.397	0.884	0.980
CT0409	854	495	717.209	768.080	4.629	0.870	0.988
CT0410	391	249	435.070	503.069	2.601	0.638	0.992
CT0411	1211	1092	1287.756	1268.637	6.645	0.932	0.984
CT0413	687	572	647.624	695.558	5.598	0.920	0.991
CT0414	740	524	645.195	710.483	5.283	0.904	0.990
CT0416	615	490	671.688	715.363	4.735	0.869	0.989
CT0429	789	495	814.709	888.280	4.959	0.932	0.985
CT0434	720	471	670.750	728.426	3.192	0.662	0.988
CT0437	915	689	861.143	942.344	3.973	0.706	0.985
CT0438	453	299	348.133	364.986	5.932	0.965	0.996
CT0456	614	409	622.787	626.006	3.258	0.694	0.990

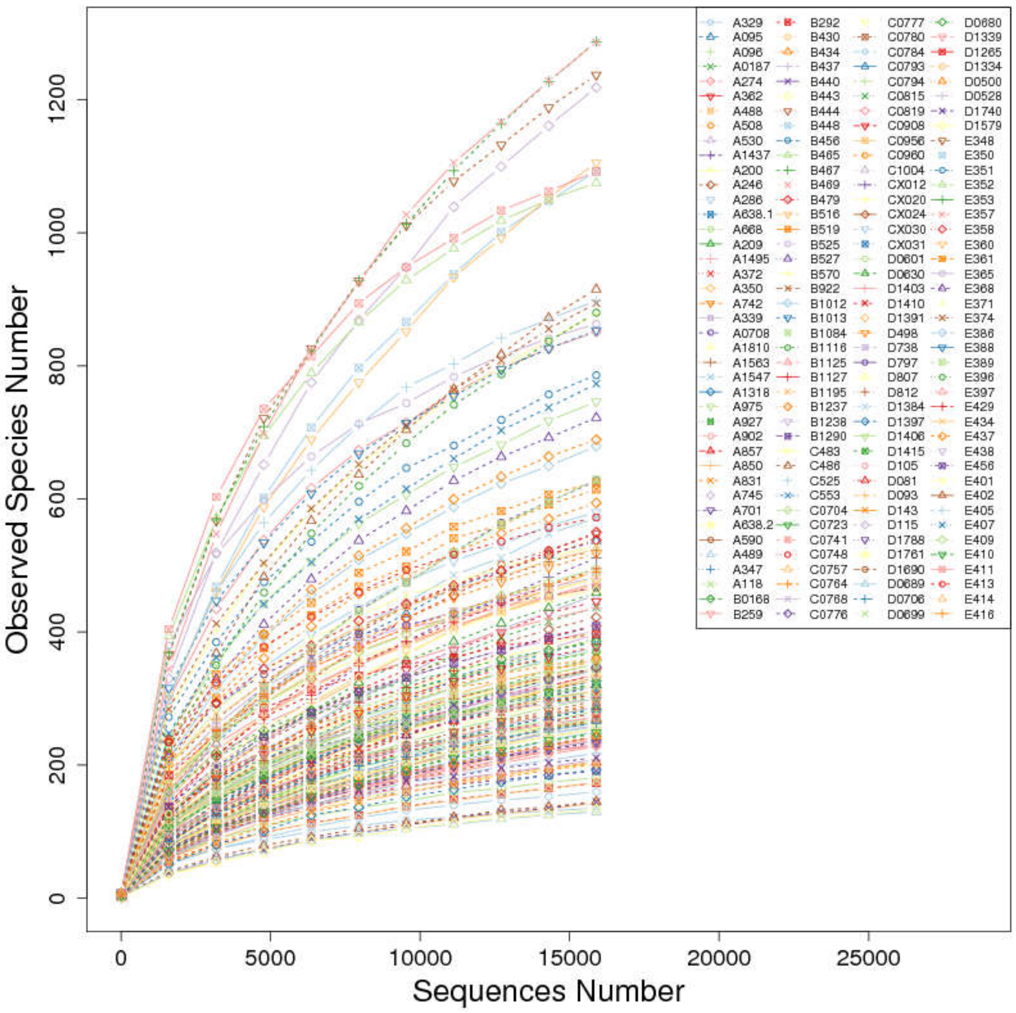

Figure 1 Rarefaction curve of all ample. A: HPHS group, *H. suis* and *H. pylori* coinfection; B: HPHF group, *H. felis* and *H. pylori* coinfection; C: HPHM group, *H. salomoni* and *H. pylori* coinfection; D: HP group, *H. pylori* monoinfection; E: CT group, *Helicobacter*-negative.
